# A cohort study of the multipollutant effects of PM_2.5_, NO_2_, and O_3_ on C-reactive protein levels during pregnancy

**DOI:** 10.1097/EE9.0000000000000308

**Published:** 2024-05-06

**Authors:** Priyanka Gogna, Michael M. Borghese, Paul J. Villeneuve, Premkumari Kumarathasan, Markey Johnson, Robin H. Shutt, Jillian Ashley-Martin, Maryse F. Bouchard, Will D. King

**Affiliations:** a Department of Public Health Sciences, Queen’s University, Kingston, Ontario, Canada; b Environmental Health Science and Research Bureau, Health Canada, Ottawa, Ontario, Canada; c School of Mathematics and Statistics, Carleton University, Ottawa, Ontario, Canada; d Water and Air Quality Bureau, Health Canada, Ottawa, Ontario, Canada; e Institut National de la Recherche Scientifique, Laval, Québec, Canada

**Keywords:** Air pollution, Inflammation, Pregnancy, Particulate matter, Greenspace

## Abstract

**Background:**

PM_2.5,_ NO_2_, and O_3_ contribute to the development of adverse pregnancy complications. While studies have investigated the independent effects of these exposures, literature on their combined effects is limited. Our objective was to study the multipollutant effects of PM_2.5_, NO_2_, and O_3_ on maternal systemic C-reactive protein (CRP) levels.

**Methods:**

We used data from 1170 pregnant women enrolled in the Maternal-Infant Research on Environmental Chemicals Study (MIREC) study in Canada. Air pollution exposures were assigned to each participant based on residential location. CRP was measured in third-trimester blood samples. We fit multipollutant linear regression models and evaluated the effects of air pollutant mixtures (14-day averages) using repeated-holdout Weighted Quantile Sum (WQS) regression and by calculating the Air Quality Health Index (AQHI).

**Results:**

In multipollutant models adjusting for NO_2,_ O_3_, and green space, each interquartile range (IQR) increase in 14-day average PM_2.5_ (IQR: 6.9 µg/m^3^) was associated with 27.1% (95% confidence interval [CI] = 6.2, 50.7) higher CRP. In air pollution mixture models adjusting for green space, each IQR increase in AQHI was associated with 37.7% (95% CI = 13.9, 66.5) higher CRP; and an IQR increase in the WQS index was associated with 78.6% (95% CI = 29.7, 146.0) higher CRP.

**Conclusion:**

PM_2.5_ has the strongest relationship of the individual pollutants examined with maternal blood CRP concentrations. Mixtures incorporating all three pollutants, assessed using the AQHI and WQS index, showed stronger relationships with CRP compared with individual pollutants and illustrate the importance of conducting multipollutant analyses.

**What this study adds:**

PM_2.5,_ NO_2_, and O_3_ contribute to the development of pregnancy complications, while green space may be protective. Studies have investigated the independent effects of these exposures, but knowledge of their combined effect is limited. We used two mixture methods to study simultaneous exposure to these air pollutants. We found that mixtures of PM_2.5_, NO_2_, and O_3_ were more strongly related to C-reactive protein in late pregnancy than any individual pollutant. No evidence for green space as a confounder was found. This work advances our current understanding of the relationship between air pollution, green space, and inflammation biomarkers in a vulnerable population – pregnant women.

## Introduction

Outdoor air pollution is associated with pregnancy complications and adverse birth outcomes, including preeclampsia,^1–3^ gestational diabetes,^4,5^ low birth weight,^6,7^ and stillbirth.^8^ This literature is primarily based on chronic PM_2.5_ (particulate matter of diameter of 2.5 μm or less) and NO_2_ (nitrogen dioxide) exposures.

Air pollution is also associated with features of the built environment, including greenness.^9–12^ Measures of green space attempt to quantify the amount of greenness near individual homes, and potentially other areas such as leisure, work, and school spaces. Surrounding green space has been positively linked to health independent of air pollution.^13,14^ Green space may improve health by reducing stress^15,16^ and increasing opportunity and motivation for outdoor physical activity.^17–19^ In addition, green space can mitigate the harmful effects of other urban exposures such as air pollution,^20^ urban heat islands,^21,22^ and noise.^23^ A large meta-analysis examining the health impacts of green space found protective effects for pregnancy outcomes such as preterm birth and small for gestational age, as well as other health effects.^14^

Increased inflammation and cortisol levels are proposed as potential intermediate mechanisms for the association between outdoor air pollution and adverse pregnancy outcomes.^24,25^ In addition, reduced inflammation may be an intermediate mechanism through which green space leads to protective health effects.^26^ C-reactive protein (CRP) is a widely studied inflammation biomarker,^27,28^ with levels reported to increase as a response to normal pregnancy, peaking in the third trimester.^29,30^ Compared with other inflammation biomarkers, the evidence for CRP as a biomarker of pregnancy complications, such as preeclampsia, gestational diabetes, and preterm birth, is more substantial.^27,28,31–34^

Studies assessing the combined effects of outdoor air pollutants and surrounding green space face methodological challenges. Exposures tend to be highly correlated with one another, and this collinearity in regression models is a potential concern when evaluating the individual effects of exposures in multipollutant models.^35–37^ Finally, differences in measurement or model accuracy for different urban exposure metrics may impact multipollutant model results.

Health effects of air pollution and green space exposures have been simultaneously assessed in several previous investigations, typically through control for coexposures as confounders or effect modifiers.^38–43^ However, few studies have assessed interactions between air pollutants and green space with respect to their associations with pregnancy outcomes,^41,44^ and to our knowledge, no studies have assessed relationships with inflammation during pregnancy. Considering that air pollution and green space exposures occur simultaneously and that outdoor air pollution mixtures are not adequately captured in single-pollutant models,^45^ there is a need for research examining joint exposure to air pollution mixtures and green space.

In previous investigations of this cohort, we showed that PM_2.5_ exposure during late pregnancy was positively associated with CRP concentrations over 14-day and 30-day exposure windows^46^ and that exposures to PM_2.5_ and NO_2_ during pregnancy were associated with reduced birthweight.^47^ The primary objective of this study is to extend the previous work on biomarkers, by investigating the independent and combined effects of PM_2.5_, NO_2_, and O_3_, on CRP concentrations. To study the independent effects, we first estimated the relationships of PM_2.5_, NO_2_, and O_3_ with CRP in multipollutant models. We also estimated the combined effects of the pollutants as a mixture. We also hypothesized that green space would attenuate associations between air pollutants and CRP, leading to weaker relationships between air pollutants and CRP in areas with higher green space. We, therefore, conducted interaction models examining green space as an effect modifier.

## Methods

### Study population and design

Data from the Canadian Maternal-Infant Research on Environmental Chemicals Study (MIREC) were used for this analysis.^48^ Women were eligible to participate in this study if they were 18 years of age or older, less than 14 weeks gestation, able to communicate in English or French, and planned to deliver at a local hospital between 2008 and 2011.^48^ In total, 2001 pregnant women were recruited during their first trimester from 10 cities in Canada and followed to delivery.^48^ CRP was analyzed in women who had a live, singleton birth and provided a blood sample during the third trimester (n = 1,573). We performed a complete case analysis, and restricted analyses to participants with complete exposure and outcome data who resided in a Forward Sortation Area (FSA, first 3 characters of Canadian postal code) less than 10 × 10 km (n = 1,170). FSA size was limited to reduce potential exposure misclassification in women residing in larger FSAs. The Canadian postal code contains six digits, with the first three digits representing FSAs, which represent well-defined geographic areas. The remaining three digits correspond to specific points in space that serve as drop-off points for mail delivery. While the FSA corresponds to a geographical area in 2D space, Canadian postal codes can be mapped to single points within FSAs but do not correspond to geographical areas.

The original study was approved by the Health Canada and Public Health Agency of Canada Research Ethics Board (file # REB 2016-017H), as well as the Research Ethics Committees of Sainte-Justine University Hospital in Montreal, Canada and all study-affiliated sites. Ethics approval for this specific analysis was obtained through Queen’s University’s Health Sciences Research Ethics Board (file no. 6030809). All participants provided informed consent.

### PM_2.5_, NO_2_, and O_3_

Differing approaches were used to derive residential exposure estimates for PM_2.5_, NO_2_, and O_3_, with the aim of generating pollutant exposures as 14-day averages before blood draw. Methods used to assign PM_2.5_ and NO_2_ exposures to MIREC participants have been described in detail elsewhere.^46,47^ Briefly, long-term PM_2.5_ and NO_2_ exposure estimates were derived from satellite data and land use regression modeling.^49,50^ Temporal resolution was added to the long-term PM_2.5_ and NO_2_ estimates by obtaining information on relative daily fluctuations in these pollutants from National Air Pollution Surveillance (NAPS) fixed-monitoring sites. This method is further described in Johnson et al, 2022. Ozone (O_3_) exposures were estimated solely from NAPS monitoring data; we assigned 24-hour mean O_3_ exposures for women in MIREC. NAPS monitors provide continuous measurements of PM_2.5_, NO_2_, and O_3_. Daily NAPS concentrations for all pollutants (PM_2.5_, NO_2_, and O_3_) were estimated for MIREC using the average daily concentrations from all NAPS sites within a 30-km radius of each participant’s FSA centroid. Pollutant exposures were assigned to participants using their residential FSA at delivery. Methods for this linkage are described elsewhere.^47^

Daily mean concentrations for all ambient air pollutants were averaged to represent exposures 14 days before the collection of blood samples for CRP measurement. In our previous work, the specific 14-day exposure window had the strongest and most consistent association with CRP levels,^46^ which is consistent with a previous review paper.^51^

### Green space

Green space exposure was assessed for each participant using green space within their FSA as a proxy for green space surrounding the home. Surrounding green space was estimated using Normalized Difference Vegetative Index (NDVI) data available through the Canadian Urban Environmental Health Research Consortium, a national data-linkage initiative for environmental exposures.^52^ The NDVI is a ratio measure of the near-infrared minus the red-light reflectance and the near-infrared plus the red-light reflectance. The NDVI ranges from −1 to +1, with higher values being associated with increased red-light absorbance, and therefore greater green vegetation in a spatial area. Values less than 0 are associated with blue space or cloud coverage and were set to zero in analyses. The NDVI is a widely used metric for measuring vegetation.^13^ Maximum annual NDVI values at the postal code level were downloaded for the years 2008–2011,^53,54^ and weighted by dwelling density to generate FSA level averages.^55^ These exposure data were then linked to MIREC participant data based on FSA, and the year of the third trimester of pregnancy for participants.

### C-reactive protein

CRP was measured in all women who had live, singleton births and provided a blood sample in their third trimester (n = 1,573). The average weeks at gestation for the blood draw was 31.6. CRP was analyzed through an affinity-based multiplex protein array, with Bio-Plex Pro Human panels (Bio-Rad, ON, Canada) and Milliplex Map kits (Millipore, Billerica, MA).^56^ The interassay and intraassay coefficients of variation for CRP were below 10%.

### Covariates

Lifestyle and demographic information was obtained from questionnaires collected throughout the pregnancy. Covariates were considered as potential confounders in our analyses if they were associated with the exposures or inflammation biomarkers,^57^ or if they were identified as a potential confounders in previous studies.^25,58,59^ The following variables were controlled for in all models: age, recruitment center (10 centers adjusted for as fixed effects^60^), income (below median Canadian household income vs. above^61^), race (White vs. non-White), self-reported prepregnancy body mass index (underweight/normal, overweight, and obese^62^), gestational weight gain (kg), alcohol consumption in early pregnancy (any vs. none), smoking status in the third trimester (never, former, quit during pregnancy, and current), physical activities in the third trimester (walking or biking hours per week), outdoor time (days with more than 3 hours spent outdoors per week in early pregnancy), season of blood draw (summer, fall, winter, and spring), and housing characteristics (furnace in home [yes vs. no], attached garage [yes vs. no], main cooking appliance used [electric only vs. electric and/or other]). Except for alcohol use, time-varying covariates measured in the third trimester were used.

### Statistical analysis

Statistical analyses were conducted using R (version 3.6.3, Vienna, Austria). CRP was right-skewed and was therefore log-transformed. All coefficients (*β*) from multivariable linear regression models were transformed using the following formula to represent the % difference in biomarker levels per interquartile range (IQR) change in each exposure: $100\times (e^{\beta }-1)$.

We used single and multipollutant models to assess the individual and independent effects of PM_2.5_, NO_2_, and O_3_ on CRP. To assess the combined effects of PM_2.5_, NO_2_, and O_3_ on CRP, we calculated a Weighted Quantile Sum (WQS) index,^63^ and calculated the Air Quality Health Index (AQHI)^64^ to transform individual pollutants variables into combined mixture variables.

WQS regression is an approach to identify key exposures that may drive associations between a mixture of environmental chemicals and health outcomes.^65^ This method models the relationship between a one-quantile increase in the entire exposure mixture and the outcome of interest under the assumptions of directional homogeneity, linearity in the relationship within quantiles of exposure and the response variable, and additivity among exposure effects.^65^ A repeated-holdout method was recently developed to mitigate unstable chemical weights and reduced study power due to data splitting for training and validation.^66^ In this procedure, data are randomly partitioned with replacement 100 times, and the WQS is repeated on each set, constraining weights to the positive direction. Bootstrapping is applied within each step to ensure index weights within each sample remain stable. We applied 100 repeated holdouts and 100 bootstraps, with a 60/40 training/validation split.^63,66^

The AQHI is a Canadian risk communication tool, developed to help provide information on air quality to Canadians. The AQHI was developed as the sum of excess mortality risk associated with individual pollutants from a time-series analysis of air pollution and mortality. The final scale is adjusted to values from 0 to 10 and calculated hourly based on trailing 3-hour average pollutant concentrations.^64^ For this analysis, the formula was applied to 14-day average estimates of PM_2.5_, NO_2_, and O_3_.

The final formula for the AQHI^64^ is:


$$\begin{array}{l} AQHI=\frac{10}{10.4}\times (100\times (e^{\left(0.000871\times NO_{2}\right)}-1+\;e^{\left(0.000537\times O_{3}\right)}-1+\;e^{0.000487\times PM_{2.5}\;}-1))\; \end{array}$$

### Effect modification analyses

NDVI was categorized into tertiles before interaction analyses. First-order interactions between each pollutant by NDVI were assessed via the inclusion of product terms in single-pollutant models in order to investigate the potential for multiplicative interactions. *P* values < 0.05 were used to assess the statistical significance of the interaction term.

### Sensitivity analyses

Analyses were also repeated by season, focusing in particular on effects during the summer months, as defined by meteorological cutoffs (June 1 to September 1).^67,68^ Seasonal differences resulting in different personal exposures to air pollutants are an important consideration in epidemiological studies.^69–73^ Personal behaviors impacting exposures to pollutants are likely to be different in warmer months, as individuals spend more time outside compared to cooler seasons, and this may lead to misclassification of exposures in epidemiology studies.^74^

## Results

Baseline characteristics in the full cohort^48^ and this analytic sample have been described previously^46^ and are provided in Table A1; http://links.lww.com/EE/A274. Briefly, participants had a mean (standard deviation [SD]) age of 32.3 (5.0), and the majority of participants were White (82.9%), had a household income above CAD$80,000, and had a college or university degree (93.0%). There were 409 unique FSAs represented in the cohort, with differences in pregnancy timing allowing for differing exposure estimates for women residing in the same FSA. Overall, 14-day mean pollutant concentrations before blood draw were as follows: PM_2.5_: 9.5 µg/m^3^ (SD: 5.0), NO_2_: 17.1 ppb (SD: 11.2), and O_3_: 24.6 ppb (SD: 7.9). The mean annual maximum NDVI for participants was 0.63 (SD: 0.1) (Table 1), which was comparable to estimates reported for the general Canadian population living in cities (mean 0.58, SD: 0.11).^75^ Pollutant concentrations were consistently higher during the summer months (Table 1). In the summer months, there was a positive and statistically significant correlation between PM_2.5_ and O_3_, which was not present in analyses of the entire year (Table 1). Other correlations were comparable between summer and the entire year. The geometric mean CRP was 16.9 mg/l (25th percentile: 0.1, 75th percentile: 35.9).

**Table 1.** Descriptive statistics for air pollution exposures and surrounding green space in MIREC participants (n = 1,170)

	Concentration					Correlation		
	Mean (SD)	25th percentile	50th percentile	75th percentile	IQR	NO_2_ (14-d average)	O_3_ (14-d average)	NDVI (annual maximum)
Overall (n = 1,170)								
PM_2.5_ (14-d average; µg/m^3^)	9.5 ± 5.0	5.6	8.7	12.5	6.9	0.33 *P* < 0.001	−0.03 *P* = 0.3	−0.28 *P* < 0.001
NO_2_ (14-d average; ppb)	17.1 ± 11.2	7.0	16.3	25.0	18.0	-	−0.40 *P* < 0.001	−0.52 *P* < 0.001
O_3_ (14-d average; ppb)	24.6 ± 7.9	18.3	24.5	30.6	12.3	-	-	0.14 *P* < 0.001
NDVI (annual maximum)	0.6 ± 0.1	0.5	0.6	0.7	0.2	-	-	-
Summer months (June–August; n = 284)								
PM_2.5_ (14-d average; µg/m^3^)	11.7 ± 5.6	7.5	11.0	15.7	8.2	0.16 *P* = 0.005	0.37 *P* < 0.001	−0.24 *P* < 0.001
NO_2_ (14-d average; ppb)	15.3 ± 8.7	7.2	16.0	22.1	14.9	-	−0.28 *P* < 0.001	−0.48 *P* < 0.001
O_3_ (14-d average; ppb)	27.4 ± 6.4	23.1	26.8	31.7	8.6	-	-	0.14 *P* = 0.02
NDVI (annual maximum)	0.6 ± 0.1	0.5	0.6	0.7	0.2	-	-	-

When comparing single-pollutant models based on the IQR of each variable, PM_2.5_ had the strongest relationship with CRP concentrations (32.3% increase per IQR change [95% confidence interval (CI) = 13.9, 53.7]) (Table 2). This relationship was slightly attenuated but remained statistically significant after adjustment for other pollutants in a multipollutant model, as well as after adjustment for annual maximum NDVI (Table 2). Each IQR increase in 14-day average PM_2.5_ was associated with 28.4% (95% CI = 8.3, 52.2) higher CRP levels after adjustment for NO_2_ and O_3_, and 27.1% (95% CI = 6.2, 50.7) higher CRP levels after additional adjustment for NDVI. Effects for NO_2_ and O_3_ were not statistically significant either before or after adjustment for NDVI (Table 2). There were no statistically significant multiplicative interactions between pollutants in multipollutant models (data not shown).

**Table 2.** Single and multipollutant models for relationships between 14-day IQR increase in PM_2.5_, NO_2_, and O_3_ with % change in CRP (n = 988)^a^

	Single-pollutant model (% change CRP^b^)	Multipollutant model not adjusted for NDVI (% change CRP^b^)	Multipollutant model with NDVI (% change CRP^c^)	Multipollutant model for summer, with NDVI (% change CRP^d^)
PM_2.5_	32.3 (13.9, 53.7)	28.4 (8.3, 52.2)	27.1 (6.2, 50.7)	68.2 (11.6, 150.9)
NO_2_	19.7 (−2, 47.7)	6.2 (−17.3, 36.3)	13.9 (−13.1, 49.2)	−27.4 (−65.7, 55.3)
O_3_	13.9 (−5.8, 39.1)	11.6 (−9.5, 37.7)	12.7 (−8.6, 39.1)	−3 (−49.8, 87.8)

a Complete case analysis was used, where participants with any missing information on exposures, outcomes, and covariates were excluded. IQR increases were as follows for each exposure: PM_2.5_: 6.9 µg/m^3^, NO_2_: 18.0 ppb, O_3_: 12.3 ppb.

b Model adjusted for: recruitment center, age, income, race, prepregnancy body mass index, gestational weight gain, alcohol consumption, smoking status, furnace, garage, main cooking appliance, outside time, physical activity, and season of blood draw.

c Model adjusted for covariates in model above as well as maximum annual NDVI.

d Model adjusted for covariates in model above as well as maximum annual NDVI. No adjustment for season.

For every IQR increase in the AQHI, there was a 37.7% increase in CRP levels (95% CI = 13.9, 66.5) after adjustment for NDVI. This estimate was higher, but less precise, in summer months (Table 3). For every IQR increase in the WQS index, adjusted for NDVI, there was a 78.6% increase in CRP levels (95% CI = 29.7, 146.0). It was not possible to run the WQS procedure for the summer months only due to the low sample size for categorical variables after the data splitting required for the procedure. Weights assigned to each pollutant through the WQS procedure were 0.28 for PM_2.5_, 0.36 for NO_2_, and 0.37 for O_3_ (Table 3). Estimates per IQR are presented in Table 3 for ease of comparability to single and multipollutant model estimates. The AQHI and WQS index mixtures had stronger relationships with CRP levels than individual pollutants in multipollutant models (Tables 2 and 3).

**Table 3.** Mixture models for relationships between PM_2.5_, NO_2_, and O_3_ as a mixture with % change in CRP (n = 988)^a^

	Mixture model not adjusted for NDVI^b^ (% change in CRP)	Mixture model with NDVI^c^ (% change in CRP)	Mixture model for summer, with NDVI^d^ (% change in CRP)
AQHI per unit increase	19.7 (6.2, 33.6)	24.6 (9.4, 40.5)	37.7 (2, 85.9)
AQHI per IQR increase	29.7 (9.4, 55.3)	37.7 (13.9, 66.5)	60.0 (3.0, 150.9)
WQS index per quartile increase	27.1 (8.3, 50.7)	33.6 (13.9, 56.8)	-
WQS index per IQR increase	61.6 (17.4, 127)	78.6 (29.7, 146.0)	-

a Complete case analysis was used, where participants with any missing information on exposures, outcomes, and covariates were excluded. IQR increases were as follows for each exposure: PM_2.5_: 6.9 µg/m^3^, NO_2_: 18.0 ppb, O_3_: 12.3 ppb. The mean, median, and IQR for the AQHI were 3.2 (SD: 1.0), 3.1, and 1.5, respectively.

b Model adjusted for: recruitment center, age, income, race, prepregnancy body mass index, gestational weight gain, alcohol consumption, smoking status, furnace, garage, main cooking appliance, outside time, physical activity, season of blood draw. WQS weights: PM_2.5_: 0.29, NO_2_: 0.31, O_3_: 0.39.

c Model adjusted for covariates in model above as well as maximum annual NDVI. WQS weights: PM_2.5_: 0.28, NO_2_: 0.36, O_3_: 0.37.

d Model adjusted for covariates in model above as well as maximum annual NDVI. No adjustment for season

Effect modification by NDVI tertiles was modeled for each pollutant separately (Table 4). Overall estimates were not appreciably different by NDVI tertile. However, effect estimates for the lowest and medium tertile of NDVI were statistically significant for PM_2.5_ (low: 33.6% change in CRP [95% CI = 9.4, 61.6] and medium: 37.7% change in CRP [95% CI = 12.2, 71.6]) and for the lowest tertile of NO_2_ (37.7% change in CRP [95% CI = 4.1, 84.0]). There were no statistically significant effects of O_3_ on CRP levels within any tertile of NDVI (Table 4).

**Table 4.** Single-pollutant and mixture models and interactions with NDVI (annual maximum) (n = 988)^a^

	Overall		Summer months	
Exposure	n	% Change CRP^b^	n	% Change in CRP^c^
PM_2.5_				
NDVI low	317	33.6 (9.4, 61.6)	90	78.6 (20.9, 163.8)
NDVI medium	332	37.7 (12.2, 71.6)	83	58.4 (10.5, 129.3)
NDVI high	339	24.6 (−0.7, 58.4)	66	37.7 (−14.8, 124.8)
		Tertile-based interaction *P* value: 0.76		Tertile−based interaction *P* value: 0.64
NO_2_				
NDVI low	317	37.7 (4.1, 84)	90	50.7 (−42.3, 289.6)
NDVI medium	332	11.6 (−16.5, 50.7)	83	−19.7 (−63.6, 78.6)
NDVI high	339	29.7 (−4.9, 80.4)	66	−15.6 (−72.7, 161.2)
		Tertile-based interaction *P* value: 0.43		Tertile-based interaction *P* value: 0.44
O_3_				
NDVI low	317	5.1 (−18.1, 36.3)	90	80.4 (−8.6, 259.7)
NDVI medium	332	20.9 (−4.9, 55.3)	83	50.7 (−17.3, 177.3)
NDVI high	339	13.9 (−10.4, 47.7)	66	107.5 (3, 322.1)
		Tertile-based interaction *P* value: 0.59		Tertile-based interaction *P* value: 0.66

NDVI tertiles: 0.00, 0.59; 0.60, 0.67; 0.68, 1.

a Complete case analysis was used, where participants with any missing information on exposures, outcomes, and covariates were excluded.

b Model adjusted for: recruitment center, age, income, race, prepregnancy body mass index, gestational weight gain, alcohol consumption, smoking status, furnace, garage, main cooking appliance, season of blood draw, outside time, and physical activity.

c Model adjusted for all covariates listed above except for season.

In the summer months, effect estimates decreased across increasing tertiles of NDVI for PM_2.5_, suggesting stronger effects of PM_2.5_ in areas with low NDVI levels (Table 4 and Table A2; http://links.lww.com/EE/A274). However, these results were only statistically significant for the lowest and medium NDVI tertile (low: 78.6% increase in CRP [95% CI = 20.9, 163.8] and medium: 58.4% change in CRP [95% CI = 10.5, 129.3]), and the overall interaction was nonsignificant.

## Discussion

### Study summary

In this study, relationships between PM_2.5_, NO_2_, and O_3_ on CRP during late pregnancy were investigated. Our findings suggest that out of PM_2.5_, NO_2_, and O_3_, PM_2.5_ has the strongest relationship with maternal blood CRP concentrations measured during late pregnancy. Green space did not appear to be an important confounder or effect modifier of this relationship in analyses. This builds on our previous results where we focused on single-pollutant models for PM_2.5_ and NO_2_.^46^ Mixtures incorporating all three pollutants, assessed using the AQHI and WQS index, showed stronger relationships with CRP compared with individual pollutants, although CIs were relatively large for mixture estimates.

There are two novel aspects to this study: the assessment of the effects of air pollutant mixtures on CRP levels during pregnancy, and the investigation of green space as a potential confounder or effect modifier of the relationships of interest. To our knowledge, no previous investigations have assessed the comprehensive effects of these exposures on inflammation during pregnancy.

### Previous studies

A few previous studies have assessed the impact of pollutant mixtures on pregnancy outcomes. A large cohort study based in California applied quantile G computation^76^ to study the combined effects of PM_2.5_, PM_10_, and NO_2_ on gestational diabetes and found that the single-pollutant and multipollutant estimates were comparable in magnitude.^77^ In contrast, we report that the mixture had a stronger effect on the outcome of interest than any individual pollutant. Consistent with our findings, other pregnancy cohort studies have reported stronger associations of indices of pollutant mixtures than individual pollutants with pregnancy outcomes such as preterm birth and intrauterine mortality.^78–80^

### Biologic plausibility

Although not consistent or statistically significant, PM_2.5_ and NO_2_ effects on CRP appeared strongest for participants with low residential surrounding green space values in this study. These analyses were exploratory and will require confirmation in future studies. We also found consistent and stronger effects for the relationships between air pollutants and CRP levels in the summer months. The effects of air pollutants may be stronger in the summer months due to increased time spent outdoors, and therefore more accurately capture estimated outdoor pollutant exposures. In addition, some toxicology studies suggest a stronger potential for ambient air pollutants to induce inflammatory responses in warmer seasons.^81,82^ This is potentially associated with temporal changes in the chemical composition of particles as well as endoxin levels.

The relationship between air pollution (individual pollutants and mixtures) and inflammation likely differs between pregnant women and the general population. Pregnancy-specific biological processes can potentially increase susceptibility to outdoor air pollution in this population.^83–85^ The vulnerability of pregnant women and fetuses to increased internal doses of environmental pollutants at the same external doses experienced by the rest of the population^83–85^ makes this subpopulation of particular interest for impactful population-level interventions. A review of particulate matter and CRP in the general population reported that many studies found no statistically significant association between PM_2.5_ and CRP, determining that overall findings were inconclusive.^51^ Two previous studies used comparable time windows as well as exposure and outcome units compared with our investigation: one reported a change in CRP per IQR increase in PM_2.5_ of 7.0% (95% CI = 1.4, 13.0),^86^ and the other of 12.0% (95% CI = −25.0, 67.0).^87^ Both studies reported effects smaller than the single-pollutant results for PM_2.5_ reported in this study.

### Limitations

The major limitations of this work include likely nondifferential exposure measurement error due to relatively large ecologic measures of exposure, and sample size for interactions. Even after the exclusion of larger FSAs, it is possible that exposure misclassification likely remains, especially considering that smaller, urban FSAs likely had larger variations in pollutant concentrations. In addition, exposure misclassification may be substantially different for PM_2.5_, NO_2_, and O_3_, and the levels of misclassification may have impacted our study findings. Specifically, NO_2_ is more spatially resolved than PM_2.5_.^88^ This may impact the interpretation of our single-pollutant models as effect estimates may be drawn closer to the null for NO_2_ compared with PM_2.5_. In a previous analysis, we quantified the degree to which nondifferential exposure misclassification for PM_2.5_ and CRP impacted effect estimates, showing a greater magnitude of effect when accounting for this misclassification.^46^ Although green space studies using NDVI have typically created buffers between 500 and 1000 m around residential areas, this was not possible given the lack of detailed residential data in MIREC.^89^ However, previous studies show a strong correlation between buffers generated at 50 m and 1000 m, and larger buffers (1000 m) appeared to be more strongly associated with health outcomes, which lends some support to our use of larger geographic units.^89–91^ Despite these observations, it is possible that the large ecologic unit in which NDVI was measured could explain our weak findings.

The stratified and interaction analyses presented in this paper were exploratory, and small sample sizes within strata limited statistical power. There were also some limitations in the use of the AQHI selected to summarize the combined effects of PM_2.5_, NO_2_, and O_3_. The AQHI was developed using data on ambient air pollution and administrative mortality data in Canada.^92^ The AQHI is calculated using trailing 3-hour averages of the concentrations of PM_2.5_, NO_2_, and O_3_. We adapted this index to reflect a 14-day average. In addition, this index weighted NO_2_ more heavily than other pollutants, while PM_2.5_ seemed to show the strongest relationship in our multipollutant models. This may explain why the AQHI estimates were weaker than estimates based on the WQS index. The use of the WQS index also had limitations. The method may overfit effects of interest because they are estimated using a supervised method, optimized to the data.^93^ Despite these limitations, the established use of the AQHI to summarize potential health impacts of poor air quality for the general public, and recent studies showing that the AQHI can be applied to acute, nonmortality effects of air pollution, make this an attractive measure to apply to this research work.^92^

Finally, the study was limited to a single measurement of CRP. Future investigations should consider longitudinal measures of CRP to assess relationships between air pollution and CRP throughout the pregnancy, in contrast with the short time window of exposure, and third trimester outcome measure used in this analysis.

## Conclusion

This study provides evidence to elucidate the complex relationships between air pollutants and surrounding green space on CRP concentrations during pregnancy. Our results highlight the importance of examining complex mixtures that humans are exposed to simultaneously, as we found that studying pollutants as a mixture showed larger effect sizes than individual pollutant effects. Although the single-pollutant approach has been useful in advancing scientific understanding of relationships, others contend that a mixture approach better reflects the reality of multiple and continuous exposures.^94,95^ Canadian air quality standards are set based on individual values for single pollutants, despite the fact that ambient pollutants represent a complex mixture.^95^ This regulatory framework is likely due to the difficulty of regulating mixtures compared to individual pollutants. With more studies linking mixture effects to specific health outcomes, it may be possible to base regulations on the combined impact of two or more pollutants. More likely, however, is the communication of health risk tools to the general public that are based on pollutant mixtures, such as the AQHI.^96^ In this work, we provide evidence for stronger effects associated with the combined mixture of PM_2.5_, NO_2_, and O_3_ (compared with individual pollutants) on inflammation levels during pregnancy. Our results suggest a greater need for considering air pollution mixtures and cumulative exposures in the context of health impact assessment.

## Conflicts of interest statement

The authors declare that they have no conflicts of interest with regard to the content of this report.

## Acknowledgments

The authors would like to acknowledge and thank the MIREC families for their interest and participation and the site and coordinating center staff for recruiting the participants and collecting and managing the data and biospecimens.

## Supplementary Material


