# Supplementary material for: A cohort study of the multipollutant effects of PM2.5, NO2, and O3 on C-reactive protein levels during pregnancy
Source: Environ Epidemiol. 2024 May 6;8(3):e308. doi: 10.1097/EE9.0000000000000308 (PMC11115979; doi:10.1097/EE9.0000000000000308)
Supplement: Supplementary file 1 [file ee9-8-e308-s001.docx]

Appendix

Table A1. Characteristics of MIREC participants and air pollution exposures (n = 1,170)

|  |  |
| --- | --- |
| **Characteristic** | **n (%)** |
| **Baseline** |  |
| **Income ($CAD)^a^** |  |
| Household < $80,000 | 414 (35.4) |
| Household >= $80,000 | 693 (59.2) |
| Don’t know/refuse to answer | 49 (4.2) |
| Missing | 14 (1.2) |
| **Education** |  |
| High-school or less | 78 (6.7) |
| College or University | 1089 (93.0) |
| Missing | 3 (0.3) |
| **Race** |  |
| White | 971 (82.9) |
| Other | 199 (17.0) |
| **Pre-pregnancy BMI** |  |
| Under/Normal weight | 705 (60.2) |
| Overweight | 227 (19.4) |
| Obese | 151 (12.9) |
| Missing | 87 (7.4) |
| **Current home type** |  |
| Single detached | 566 (48.3) |
| Duplex or townhouse | 339 (28.9) |
| 100% residential building | 246 (21.0) |
| Mix residential and commercial | 18 (1.5) |
| Missing | 1 (0.1) |
| **Attached garage** |  |
| Yes | 430 (36.8) |
| No | 740 (63.3) |
| **Type of cooking appliances used** |  |
| Electric stove only | 388 (33.2) |
| Electric and other (*gas stove, wood stove, charcoal BBQ, propane/gas BBQ)* | 781 (66.8) |
| Missing | 1 (0.1) |
| **Furnace in home** |  |
| No | 440 (37.6) |
| Yes | 689 (59.0) |
| Missing | 41 (3.5) |
| **Alcohol Consumption** |  |
| No consumption | 951 (81.3) |
| Any consumption | 218 (18.6) |
| Missing | 1 (0.1) |
| **Smoking status** |  |
| Never | 730 (62.4) |
| Former | 305 (26.0) |
| Quit During This Pregnancy | 87 (7.4) |
| Current | 48 (4.0) |
| **Season of Blood-draw** |  |
| Winter | 263 (22.5) |
| Spring | 334 (28.6) |
| Summer | 284 (24.3) |
| Fall | 289 (24.7) |
| **Continuous Characteristics** | **Mean ± SD** |
| **Age (years)** | 32.3 **±** 5.0 |
| **Gestational Weight Gain (kg)** | 15.4 **±** 5. 9 |
| **Activity (hours per week)** | 17.8 **±** 15.8 |
| **Outside time (days spent outside for ≥ 30 minutes between 9AM-4PM in past month)** | 13.7 **±** 10.3 |

MIREC – Maternal-Infant Research on Environmental Chemicals Study, BMI – body mass index,

^a^ Income cut-off chosen based on income threshold for two-parent Canadian household.

Table A2. Multipollutant Models for relationships between 14-day IQR increase in PM_2.5_, NO_2_, and O_3_, and IQR increase in annual maximum NDVI with CRP Stratified by Season (n = 988)^a^

| **Pollutant** | **CRP (Fall)**  **% Change^b^** | **CRP (Winter)**  **% Change^b^** | **CRP (Spring)**  **% Change^b^** | **CRP (Summer)**  **% Change^b^** |
| --- | --- | --- | --- | --- |
| PM_2.5_  (per IQR increase in 14-day exposure) | 25.9 (-12.2, 80.4) | 0.7 (-40, 68.2) | -14.8 (-46.7, 36.3) | 68.2 (11.6, 150.9) |
| NO_2_  (per IQR increase in 14-day exposure) | 23.4 (-32.3, 124.8) | 13.9 (-37.5, 105.4) | 18.5 (-33.6, 113.8) | -27.4 (-65.7, 55.3) |
| O_3_  (per IQR increase in 14-day exposure) | 6.2 (-30.2, 63.2) | 13.9 (-32.3, 89.6) | -23.7 (-53.2, 24.6) | -3 (-49.8, 87.8) |

^a^ Complete case analysis was used, where participants with any missing information on exposures, outcomes, and covariates were excluded.

^b^ Models adjusted for: Recruitment centre, age, income, race, pre-pregnancy BMI, gestational weight gain, alcohol consumption, smoking status, furnace, garage, main cooking appliance, outside time, physical activity, and NDVI
